# Exfoliated Ferrierite-Related Unilamellar Nanosheets in Solution and Their Use for Preparation of Mixed Zeolite Hierarchical Structures

**DOI:** 10.1021/jacs.1c04081

**Published:** 2021-07-15

**Authors:** Wieslaw J. Roth, Takayoshi Sasaki, Karol Wolski, Yasuo Ebina, Dai-Ming Tang, Yuichi Michiue, Nobuyuki Sakai, Renzhi Ma, Ovidiu Cretu, Jun Kikkawa, Koji Kimoto, Katarzyna Kalahurska, Barbara Gil, Michal Mazur, Szczepan Zapotoczny, Jiri Čejka, Justyna Grzybek, Andrzej Kowalczyk

**Affiliations:** † Faculty of Chemistry, Jagiellonian University, Gronostajowa 2, Kraków 30-387, Poland; ‡ International Centre for Materials Nanoarchitectonics (WPI-MANA), National Institute for Materials Science (NIMS), 1-1 Namiki, Tsukuba 305-0044, Ibaraki, Japan; § Department of Physical and Macromolecular Chemistry, Faculty of Science, Charles University, Hlavova 8, Prague 2 12840, Czech Republic; ∥ Research Center for Advanced Measurement and Characterization, National Institute for Materials Science, 1-1 Namiki, Tsukuba 305-0044, Japan

## Abstract

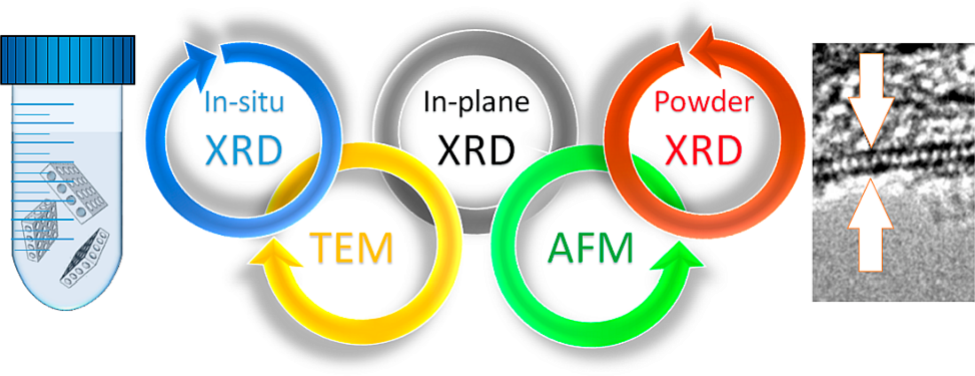

Direct exfoliation of layered zeolites into solutions of monolayers has remained unresolved since the 1990s. Recently, zeolite MCM-56 with the MWW topology (layers denoted mww) has been exfoliated directly in high yield by soft-chemical treatment with tetrabutylammonium hydroxide (TBAOH). This has enabled preparation of zeolite-based hierarchical materials and intimate composites with other active species that are unimaginable via the conventional solid-state routes. The extension to other frameworks, which provides broader benefits, diversified activity, and functionality, is not routine and requires finding suitable synthesis formulations, viz. compositions and conditions, of the layered zeolites themselves. This article reports exfoliation and characterization of layers with ferrierite-related structure, denoted bifer, having rectangular lattice constants like those of the FER and CDO zeolites, and thickness of approximately 2 nm, which is twice that of the so-called fer layer. Several techniques were combined to prove the exfoliation, supported by simulations: AFM; in-plane, in situ, and powder X-ray diffraction; TEM; and SAED. The results confirmed (i) the structure and crystallinity of the layers without unequivocal differentiation between the FER and CDO topologies and (ii) uniform thickness in solution (monodispersity), ruling out significant multilayered particles and other impurities. The bifer layers are zeolitic with Brønsted acid sites, demonstrated catalytic activity in the alkylation of mesitylene with benzyl alcohol, and intralayer pores visible in TEM. The practical benefits are demonstrated by the preparation of unprecedented intimately mixed zeolite composites with the mww, with activity greater than the sum of the components despite high content of inert silica as pillars.

## Introduction

We have recently reported a breakthrough in delamination/exfoliation of layered zeolites by showing direct high yield exfoliation of the zeolite MCM-56 with MWW topology into dispersions of 2.5 nm thick monolayers in a liquid upon treatment with tetrabutylammonium hydroxide solutions (TBAOH).^1^ This soft-chemical process^2−4^ is one of the most effective delamination procedures that presents many practical benefits, unfeasible with bulk solids, for designing and synthesis of various materials using zeolite monolayers in solutions/liquid dispersion as building blocks.^5−8^ Fundamentally, a complete exfoliation represents the ultimate manifestation of the two-dimensional (2D) nature for layered solids.^9^ It has not been shown and proven conclusively for 2D zeolites until now.^7,10−14^ The tentative explanation for this lack of genuine high yield exfoliation is that suitable synthesis formulations (gel compositions), presumably giving low level of intergrowths, are needed for sufficiently high efficiency and yield of exfoliation, which in practice is very simple to carry out. This goal has been pursued previously by various strategies^15−21^ since the discovery of layered zeolites three decades ago,^22−24^ but no comparable outcomes, i.e., direct soft-chemical exfoliation in high yield has been documented.^10,12,25^ At this point, the extension of this convenient and facile exfoliation to other zeolites depends on finding suitable synthesis formulations. This article reports the second example of such a material. It has ferrierite-related structure with approximately 2 nm layer thickness. The complete proof of exfoliation into unilamellar nanosheets consists of two parts: confirmation of monodispersity in solution, i.e., isolated monolayers of uniform thickness, and structure of the layers in solution and upon isolation as a solid with evidence of preserved of crystallinity. To this end, a suite of five characterization methods provides sufficient evidence.^1^ Monodispersity is demonstrated by AFM and in situ XRD of the colloidal sample, which also confirms the layer structure in solution. Separately, the structure is characterized by in-plane XRD, TEM, and powder XRD of reassembled layers, which provide detailed information about crystallographic dimensions and preserved layer integrity. These results are complemented by characterization of products from reassembled layers, alone or in combination with other compounds and components, which also demonstrates the practical side and pathways to synthesize functional materials.

The present layered material, designated Al-ZSM-55, was obtained by modification of the synthesis procedure of a layered zeolite precursor, ZSM-55, which is composed of 0.9 nm thick ferrierite layers (designated fer with lower case letters as proposed by Marler et al.;^26^ the capital letters like MWW will be retained for topologies, whereas the corresponding layer will be denoted mww). The synthesis modification involved substitution of Al instead of B in the procedure for making of ZSM-55, which includes choline as the structure-directing agent.^27^ The fer layers in ZSM-55 are related by translation and produce zeolite CDO with unidimensional eight-member-ring (MR) channels upon topotactic condensation at high temperature.^28^ The alternative stacking of fer layers, by reflection in mirror planes, produces zeolite FER with alternating 10- and 6-MR channels in cross section, and perpendicular 8-MR channels.^28−30^ The FER and CDO structures can be interconverted, which in practice is achieved before calcination by inducing lateral layer shifts (by 1/2 *b*)^30,31^ through intercalation and pH adjustment.^28,31,32^ Additionally, the interlayer space between stacked fer layers can be expanded by intercalation of organic molecules, including surfactants affording *d*-spacing above 3 nm.^33,34^ The applied synthesis modification, substitution of B with Al, resulted in layers with doubled layer thickness, i.e., ca. 2 nm. This made things more complicated than with the original case of MCM-56, because the exact layer structure had to be treated as unknown and requiring determination. Its close relation to the fer layer structure was confirmed (vide infra), and because it is twice as thick, it is designed here as bifer for convenience and until the structure is fully elucidated.

The availability of two types of layers, mww and bifer, in solutions enabled unprecedented opportunities, impossible with bulk layered materials, namely, preparation of intimate mixtures of zeolite monolayers and generation of hierarchical structures combining activities of different frameworks. This was carried out as an illustration of this potential by combining both solutions and isolation of mixed zeolite solids from the homogeneous liquid. The flocculation was carried out by the addition of the cationic surfactant hexadecyltrimethylammonium (HDTMA), and after treatment with tetraethylorthosilicate (TEOS) and calcination a pillared layered structure combining both mww and bifer layers was obtained. This showed that solutions of exfoliated zeolite layers can be used to prepare unprecedented materials with mixed, hierarchical, and other advanced structures and compositions.

## Results and Discussion

Monolayer solutions were obtained as described previously with MCM-56 in one or two steps by reacting solid Al-ZSM-55 samples with up to 10% w/w TBAOH solutions (typically 0.5 g solid and 15–30 mL solution) and purification (removal of larger particles) by centrifugation at 10 000 rpm for 10 or more minutes.^1^ The solutions were visibly translucent with yields up to 70% w/w layers in solution vs the amount of the starting solid and typical concentrations equal to 1–2% (weight layers/weight solution).

### Characterization of Layer Thickness and Monodispersity by AFM

**Figure 1:**
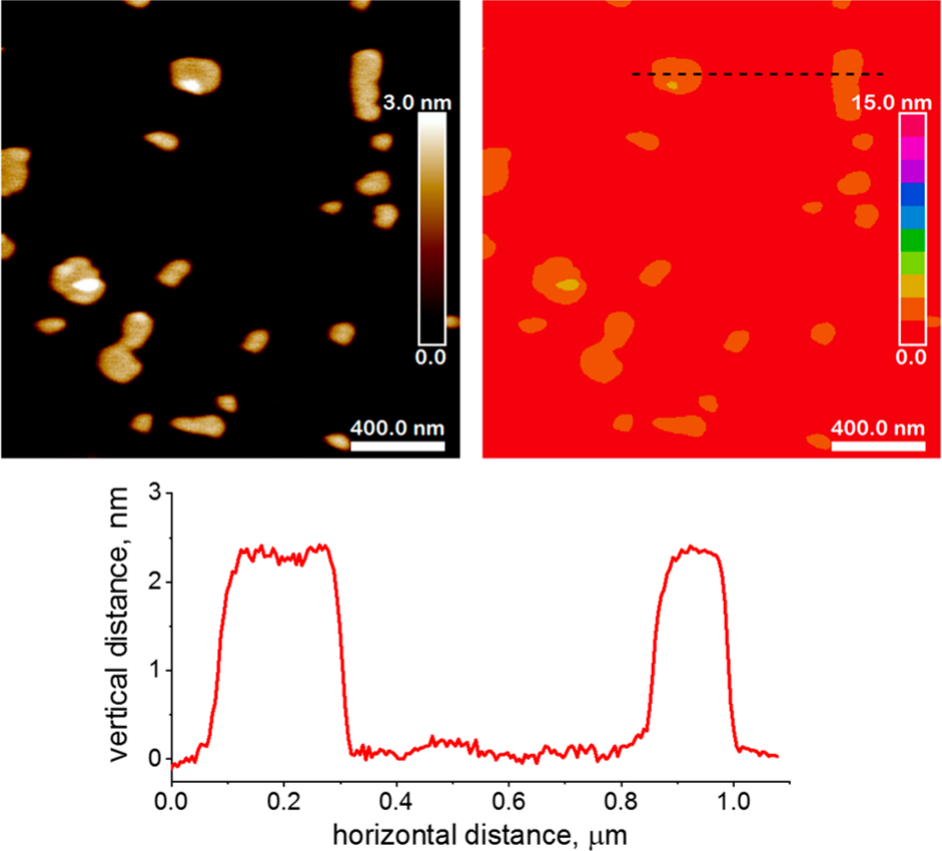
AFM topography images of the layers deposited from TBAOH solutions of Al-ZSM-55 represented in the classical (on the left side) and zone-type scale (on the right side) views with cross-section profiles extracted from the location marked by the dashed black line.

The solutions of exfoliated layers obtained by the above procedure were diluted 100-fold for AFM visualization. The images showed flat sheets (bifer layers) with thickness 2.1 ± 0.1 nm and lateral dimensions up to about 300 nm (see Figure 1). The analysis conducted in eight different places (data extracted from eight 5 × 5 μm^2^ AFM images) showed the estimated content of the bifer unilamellar nanosheets to be about 94%, whereas the remaining 6% was associated with multilayer structures (Figure S1). The determined layer thickness, 2.1 nm, represents hydrated bifer layers.

### 2D Cell Dimensions by in-Plane XRD

**Figure 2:**
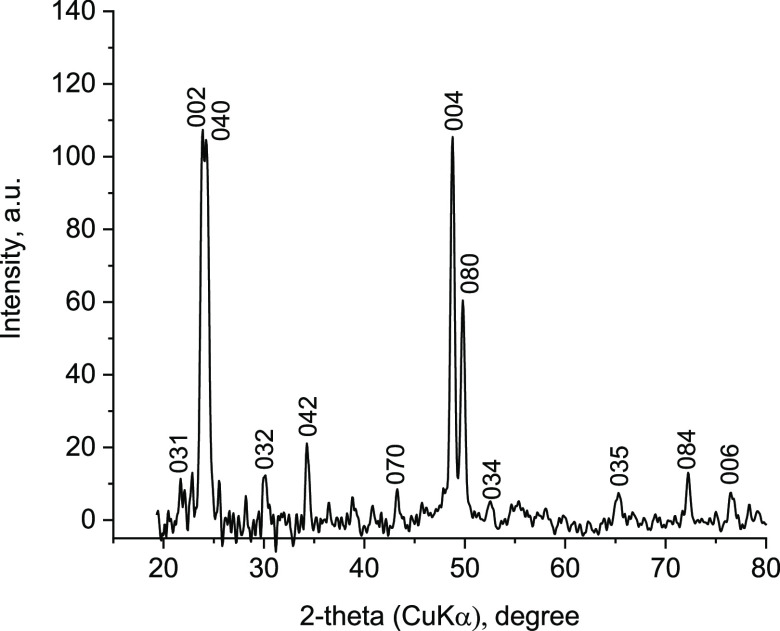
In-plane XRD pattern of the bifer nanosheets Langmuir–Blodgett film, after the baseline removal and selecting peaks having fwhm ∼0.2° by applying *Appleman* software.^35^ The 2θ axis was recalculated for CuKα radiation λ = 0.154056 nm from the original 0.11988(2) nm.

AFM informs only about the presence of monolayers, which must be characterized structurally, e.g., by unit-cell determination. This was carried out in two steps. First, the planar unit cell was determined based on in-plane XRD from a monolayer film deposited on a Si substrate. Second, the bifer layer thickness was estimated from the powder XRDs of restacked and calcined layers. The in-plane pattern obtained after baseline subtraction revealed a series of sharp peaks, shown in Figure 2, proving the crystalline (periodic) nature of the layers. All peaks could be indexed based on a 2D rectangular unit cell with dimensions, listed in Table 1, that matched closely the lattice constants of both FER and CDO structures from the IZA Structure Commission database.^36^ It must be noted that the FER and CDO data as well as the experimental powder XRDs discussed later correspond to restacked layers so there may be a slight real difference, other than experimental error, from the nanosheets investigated by in situ XRD, i.e., unilamellar without stacking.

**Table 1:** Comparison of the Unit-Cell Dimensions in Nanometers of bifer layers (*b* and *c* Axes) and Restacked Layers (*a* Axis) with related Materials Based on Fer Layers^a^

unit-cell axes	bifer	FER	CDO	ZSM-55 related
*a*-uncalcined	>2.6 (lyophilized)			2.1235
*a*-calcined	1.76	1.9018	1.87151 (b)	1.82857
*b*	1.4638(5)^b^	1.4303	1.40986 (c)	1.38034
*c*	0.7461(2)^b^	0.7541	0.755566 (a)	0.7433

a All values were obtained for multilayered structures except the bifer as indicated. The *a*, *b*, *c* in parentheses refer to axis designations in the original sources.

b Single layer.

The similarity of all *b* and *c* cell constants strongly suggests close structural relationships among them, although it does not prove that the bifer layer consists of two fused fer layers. Still, this is adopted here as a working model that is needed, for example, for in situ XRD calculations. This model is subsequently shown as highly probable based on the available evidence. It must be viewed as subject to revision and needing more direct evidence, which may require a separate specialized study. It is not possible to differentiate between the possible FER or CDO structures, which themselves are practically indistinguishable based on the unit cell values. As seen below, this “too close to call” for distinguishing between the FER and CDO structure of the bifer layer model persists in all subsequent characterizations and simulations. To examine alternative possibilities, we have also searched the IZA database for comparable unit cell dimensions of 200 most common topologies and did not find a reasonably matching set of these two values (1.4 and 0.75 nm, ± 0.1 nm). It does not completely rule out the possibility of some unknown internal structure of the bifer layer but strongly tilts toward FER or CDO as the most probable options and assuming growth without internal defects.

### Layer Thickness and Structure by Powder XRD

**Figure 3:**
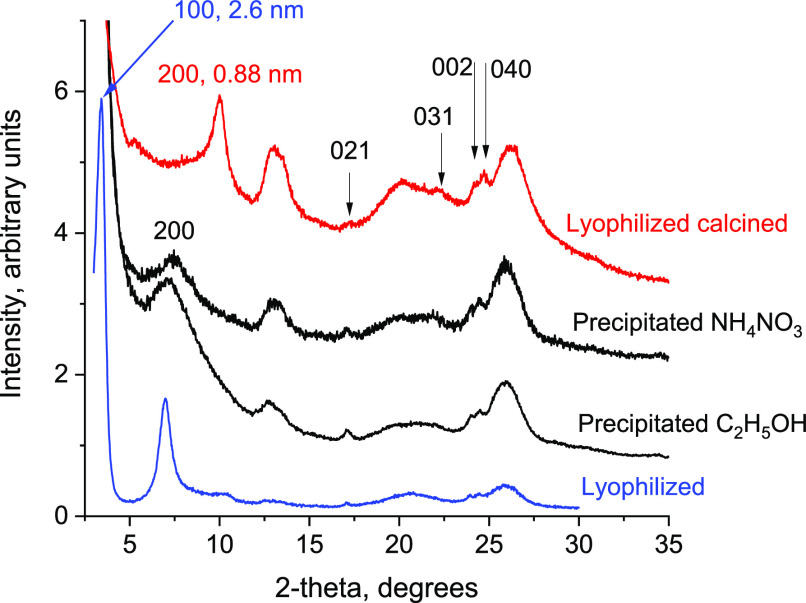
XRD patterns of bifer layers recovered from solutions as powders by lyophilization and precipitation with ammonium nitrate and ethanol. The intensities were adjusted individually for better visibility of the identifying features.

The evaluation of the third crystallographic dimension, layer thickness, and the overall structure characterization has been carried out by powder XRD (of restacked layers). The thickness of a dehydrated (calcined) bifer layer is calculated from the interlayer 200 reflection as equal to ca. 1.8 nm. To start with, a correlation between the in-plane XRD discussed above and the powder XRD is established first based on the indexed intralayer reflections, see Figure 3 and Figures S2–S5 and Table S1. The doublet at 23.9–24.4 ± 0.1° 2θ in the powder XRD with *d*-spacings 0.372 and 0.365 nm can be identified as the 002 and 040 reflections, matching two prominent peaks at the same (recalculated) position in the in-plane XRD. Additional less prominent and not always distinct intralayer reflections in powder XRD have indices 021 (outside the in-plane range) and 031 at approximately 17 and 22 (0.521 and 0.404 nm). The doublet 002 and 040 is particularly noticeable and has played the role of a fingerprint for detecting the presence of bifer layers in general. For example, it helped to establish a close relationship between the present bifer materials and the aluminosilicate patented in 1980s designated FU-1.^37,38^

Following the confirmation of the intralayer features and dimensions in the powder XRD, the layer thickness can be estimated from the patterns of calcined (restacked) bifer materials. They show a low angle peak at 0.88 nm (10.0° 2θ CuKα radiation) that can be assigned to the 200 reflection. It should be noted first that often the scattering intensity and quality of the XRD pattern diminishes upon calcination, suggesting packing disorder and possible partial degradation. More consequentially, the stacking of layers upon condensation occurs most likely without pairing of OH groups from opposite layers to produce a more complete framework with Si–O–Si interlayer bridges. The calcination product is most likely the so-called subzeolite material showing interlayer distance shorter than in a (hypothetical) complete and ordered framework, as observed with NSI, FER, and other zeolites.^39^ So, the actual vertical dimension of the layer including OH groups on the surface is assumed to be longer than 1.76 nm calculated from the 200 peak position and it is most likely similar to the CDO and FER unit cell values, again suggesting analogous structure.

The uncalcined bifer materials isolated by lyophilization also show the expected intralayer 0*kl* reflections discussed above but the interlayer distance is significantly expanded to 2.6 nm and higher. It must include the layer thickness (1.8 nm) and expansion due to intercalated TBA cations and water. The interlayer reflections appear in a series of up to three peaks at roughly 3, 6, and 9° 2θ CuKα radiation, as orders of the interlayer distance, which is slightly variable with different samples (Figure S2). This assignment is corroborated by heating up to 540 °C revealing gradual contraction of the interlayer spacing (Figure S3 and Table S1). Starting from the sample heated to 250 °C, the 200 peak becomes the most intense. The observed variability is typical for 2D solids and results from differences in hydration and packing of organic guest molecules. In organic intercalated derivatives (vide infra), the interlayer reflections appear at positions that depend on and reflect the size and content of guest molecules (Table S2).

In addition to the distinct reflections, the XRD patterns also contain broad scattering, especially at ca. 13, 22, and 27° 2θ CuKα radiation, which are most likely due to small crystal/layer dimensions, internal disorder, or both. The distinct broad scattering at 13° 2θ may be related to two intralayer peaks near 12–13° 2θ in the FER and CDO patterns (020 and 011) but we have not found a direct way to correlate/explain this feature for the bifer layer.

### In Situ XRD: Confirmation of Unilamellar Dispersions and the Layer Structure

In situ XRD measurements provide information about layers in solution: uniform thickness, if applicable, and structure, as has been demonstrated with various dispersions of 2D nanosheets of metal oxides and hydroxides.^40−45^ In this method, the experimental profile is compared to the square of the layer structure factors, which should correspond to the scattering from the aggregate of 2D crystallites that lie parallel to the XRD sample holder. This technique was also crucial for confirmation of the first direct layered zeolite exfoliation into solution (MCM-56).^1^

**Figure 4:**
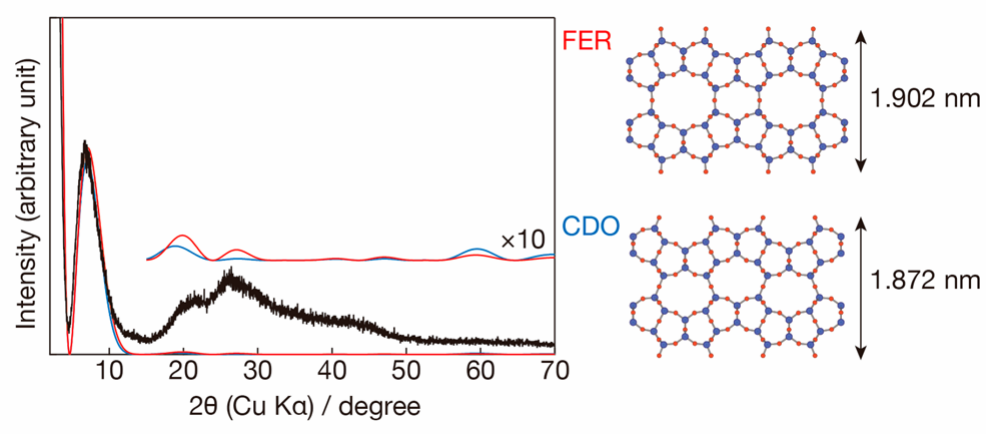
In situ XRD data at 95% relative humidity of the colloidal bifer aggregate recovered from the dispersion via high-speed centrifugation. Red and blue traces represent the square of the structure factor calculated for the FER and CDO structures consisting of two fer layers, respectively.

The in situ XRD data, measured at a relative humidity of 95% to prevent drying, gave a continuous and wavy profile shown in Figure 4. The calculated patterns were obtained for both FER and CDO topologies, based on squares of the structure factors, and showed a good match to the experimental data, particularly in the 2θ angle range below 10°. The calculated and experimental profile at the higher angular range also appeared similar, except for the intensity difference. Interpretation of the data in the 20–50° 2θ range is rather difficult because the presence of water contributes a large hump.

Structure factor plots were also calculated for single fer layers (ca. 0.9 nm thick) but turned out to deviate considerably from the experimental XRD pattern (Figure S6). In contrast, the FER and CDO topologies show a good fit between calculated and experimental data but the differences are not sufficient to favor one of these structures. It is true that calculations indicate some intensity differences between both structures in the 20–30° 2θ range but it is not significant enough to consider one topology as more likely. On the other hand, the observed similarity with calculated profiles reduces the chances for an alternative non-ferrierite-related structure of the obtained bifer nanosheets.

### TEM Imaging

The TEM study combined several types of determinations: low-magnification view of the nanosheets, selected area electron diffraction (SAED), and cross-sectional and top view image of the nanosheets. The results confirmed the basic structural features of the layers from the preceding techniques, namely, their crystalline/periodic structure and cell dimensions. In addition, intralayer channels consistent with both CDO and FER frameworks were observed.

**Figure 5:**
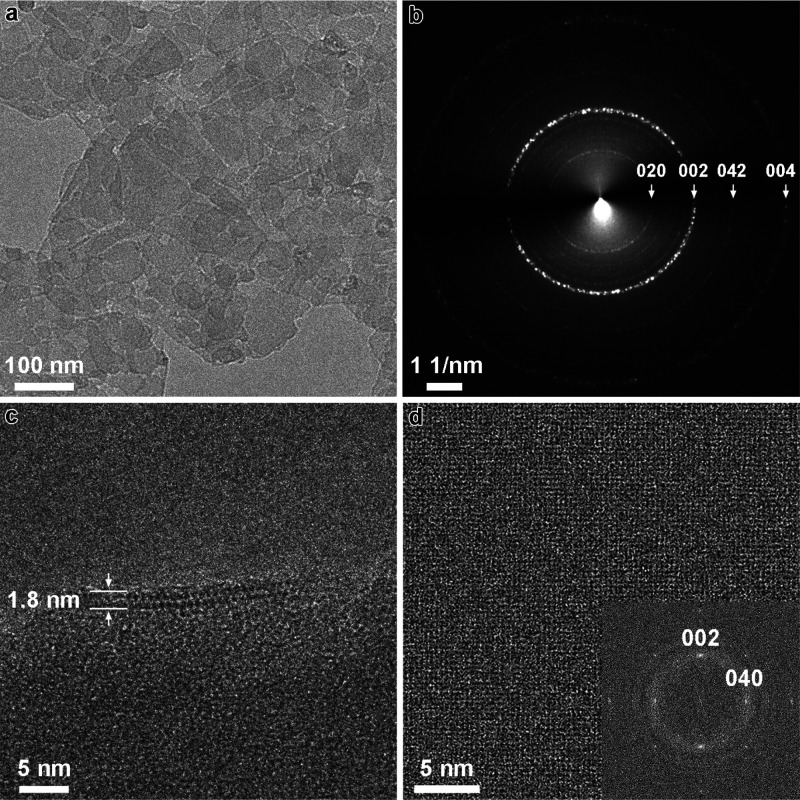
TEM characterizations. (a) Low-magnification TEM image showing overlapped nanosheets of around 100 nm in dimension. (b) SAED pattern with concentric rings indexed according to the in-plane diffraction. (c) Cross-sectional TEM image showing layered structure with the thickness around 1.8 nm. (d) High-resolution in-plane TEM image revealing homogeneous lattices corresponding to 002 and 040 reflections in fast Fourier transform (FFT) pattern.

A low-magnification TEM image (Figure 5a) shows many nanosheets with the lateral dimensions around 100 nm, assembled into a continuous film. This film is homogeneous, and its weak contrast indicates uniform and small thickness of the nanosheets. The SAED pattern (Figure 5b) shows concentric rings which could be indexed mostly as in-plane reflections of the nanosheets. The most intense ring, marked 002, corresponds to the “fingerprint” doublet discussed above and probably includes the 040 reflection as well, since they will be difficult to resolve. Expanded SEAD analysis including weak reflections is provided in Figure S7 and Table S3. The cross-section view of a nanosheet (Figure 5c) shows a porous layer with thickness around 1.8 nm, consistent with the AFM and powder XRD. The layer contains a row of uniform pores seen as white features. This image does not allow more detailed measurement of the pore spacing but a remarkably distinct image was obtained with a mixed MWW/bifer sample (vide infra). It shows the spacing between pores of ca. 0.70–0.74 nm, which can be either CDO along the *b* or *c* axes or FER along *b* axis. Edge-on TEM images with sufficient high quality enabling comparison with the atomic model and simulated TEM images (Figure S8) showed a face-centered pattern with CDO along [001] zone axis or FER along [010] zone axis. Another side-view showed the pores arranged into a rectangle pattern, that is consistent with FER along [001] along zone axis. This supports the FER-like option for the layer structure, but it is still viewed as tentative needing further validation for the bulk. A high-resolution TEM image of the in-plane structure is demonstrated in Figure 5d. Average background subtraction filter (ABSF) is used to enhance the contrast of the crystalline structure.^46^ Clear crystalline lattices could be seen over a large area up to tens of nanometers, revealing high degree of crystallinity. A fast Fourier transform (FFT) pattern showed perpendicular reflections with assigned indices (002) and (040) of the doublet observed in the XRD at ca. 24° 2θ.

### Zeolitic Nature of the bifer Layers and the Mechanism of Formation

The preceding results confirmed monodispersity and structure of the layers, as far as it can be achieved at this point. True zeolitic nature of the bifer layers was proven by FTIR. It revealed features typical for a zeolite in the hydroxyl region showing the characteristic strong Si–OH–Al band corresponding to Brønsted acid sites. The overall acid site concentration, determined by ammonia adsorption, was around 450 μmol/g, which corresponds to estimated Si/Al = 36. The concentrations obtained by adsorption of pyridine and pivalonitrile were lower, 150 and 120 μmol/g, respectively, and correspond to acid sites on the surface, as both molecules do not enter pores of both CDO and FER. The recovered layers also showed relatively high catalytic activity in the benzylation of mesitylene as a test reaction for activity toward bulky molecules characterizing more open porous solids (Figure S10).

The performed physical characterizations have not allowed definite differentiation between FER and CDO as possible structures of the bifer layers, but there are chemical-synthetic arguments, which justify proposal of the most likely option, namely FER. The possibility of faulted (misaligned) connection of two fer layers is less likely because of regular channels in the layer observed in TEM. The first clue favoring the FER structure is the fact that bifer layers are formed in the presence of Al in the synthesis of ZSM-55, in contrast to B, which results in formation of a single fer layer structure (ZSM-55). As postulated elsewhere, based on preferential formation of layered zeolites in siliceous systems, Al atoms on a layer surface seem to promote framework-like 3D connectivity rather than termination as a layer.^47^ This can explain formation/growth as fused bilayer, bifer, instead of isolated fer layers. As for the mode of fusion, FER is favored both because of Al presence and the fact that CDO framework has not been synthesized directly, only via its layered precursors. CDO precursors do not form in the presence of added Al in alkali-based synthesis, only with F as the mineralizer. On the basis of these chemical precedents, the most likely internal structure of bifer is proposed to be FER.

### Reassembly of the bifer Nanosheets into Intercalated Layered Materials

Bifer nanosheets dispersed in solutions have been reassembled as composite solid materials by treatment with suitable organic compounds. This has been carried out to complement the physical characterization and to demonstrate practical potential for designing and synthesis of new materials. The layers are negatively charged and react readily with quaternary ammonium compounds, producing multilayered intercalated structures.

**Figure 6:**
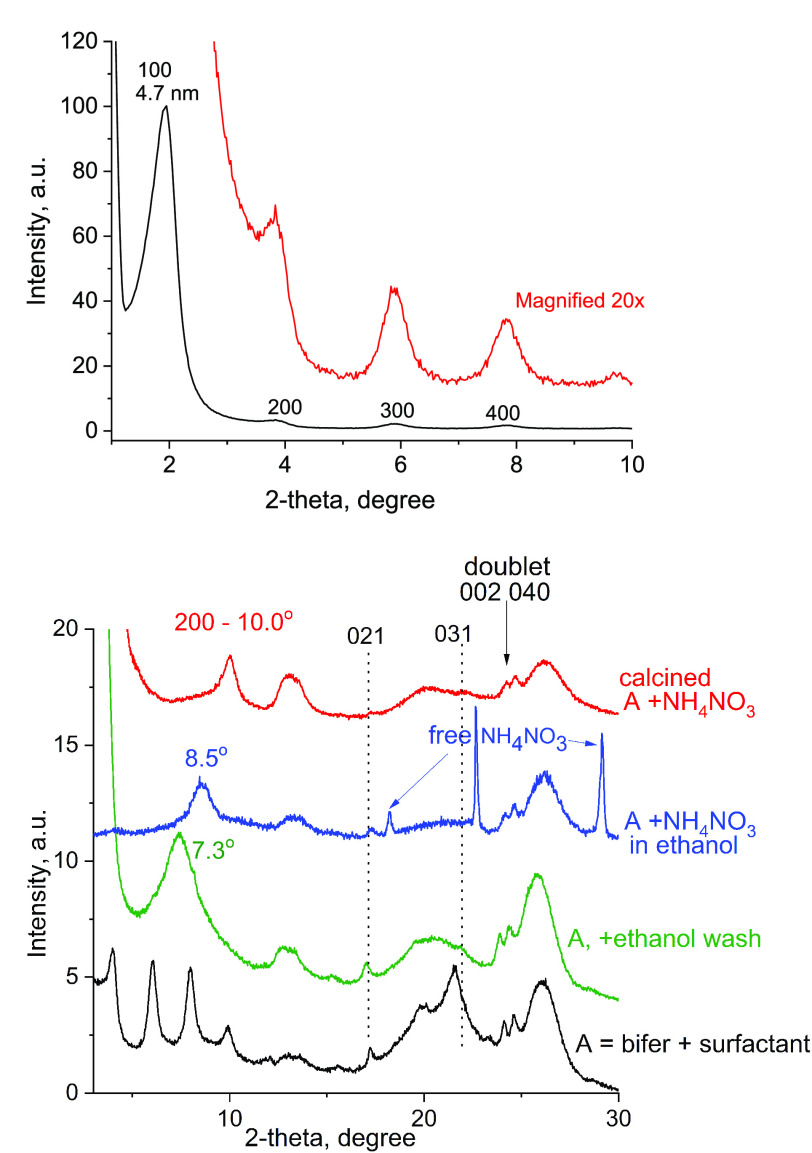
XRD pattern of the bifer layers flocculated with surfactant HDTMA^+^, designated ‘A’, and derivative materials. Top–low angle interlayer reflections, bottom–higher angle scans.

Surfactant cation, hexadecyltrimethylammonium (HDTMA) flocculates bifer nanosheets from a solution, producing multilayered composites with regular stacking (Figure 6), indicated by the relatively sharp 100 reflection at 4.7 nm with up to the fourth order peak, as shown in Figure 6. This distance corresponds to approximately 2.7 nm expansion with the surfactant bilayer separating bifer layers and the product is equivalent to a surfactant swollen layered (bifer) material. The signature intralayer reflections identifying the bifer layer, at ca. 17° 2θ and the doublet at 24° 2θ, can be also discerned. They become more visible upon removal of the surfactant under mild conditions (ethanol wash or exchange with ammonium nitrate in ethanol (Figure 6, bottom, pattern second from the bottom), which is accompanied by structural contraction (*d*-spacing shifting to 6.5–8.5° 2θ). The final product obtained upon calcination shows relatively high quality XRD in which the intralayer reflections are again quite distinct and the apparent interlayer 200 reflection is rather well-defined with a maximum at 10° 2θ (1.76 nm interlayer repeat).

Comparable materials were obtained with other cations, e.g., tetraethylammonium hydroxide (TEA–OH), giving intercalated composite with *d*-spacing of ca. 3.1 nm, consistent with the layer thickness (2 nm) and the size of TEA^+^. The XRD after calcination was similar to the HDTMA derivative showing the 200 peak at 10° 2θ with relatively high quality.

### Mixtures of Zeolite Nanosheets with mww and bifer Topologies

Solutions of zeolite monolayers with different topologies can be combined to prepare previously impossible composite materials consisting of intimately mixed zeolite nanosheets. Such mixed zeolites possess hierarchical structure and can combine functionalities of both frameworks, such as MWW and MFI (as versatile active zeolites available in layered forms), with increased open space and other activity/structure features. The currently available mww and bifer layers in solution have been used to explore synthesis and properties of such materials as an illustration of this potential.

**Figure 7:**
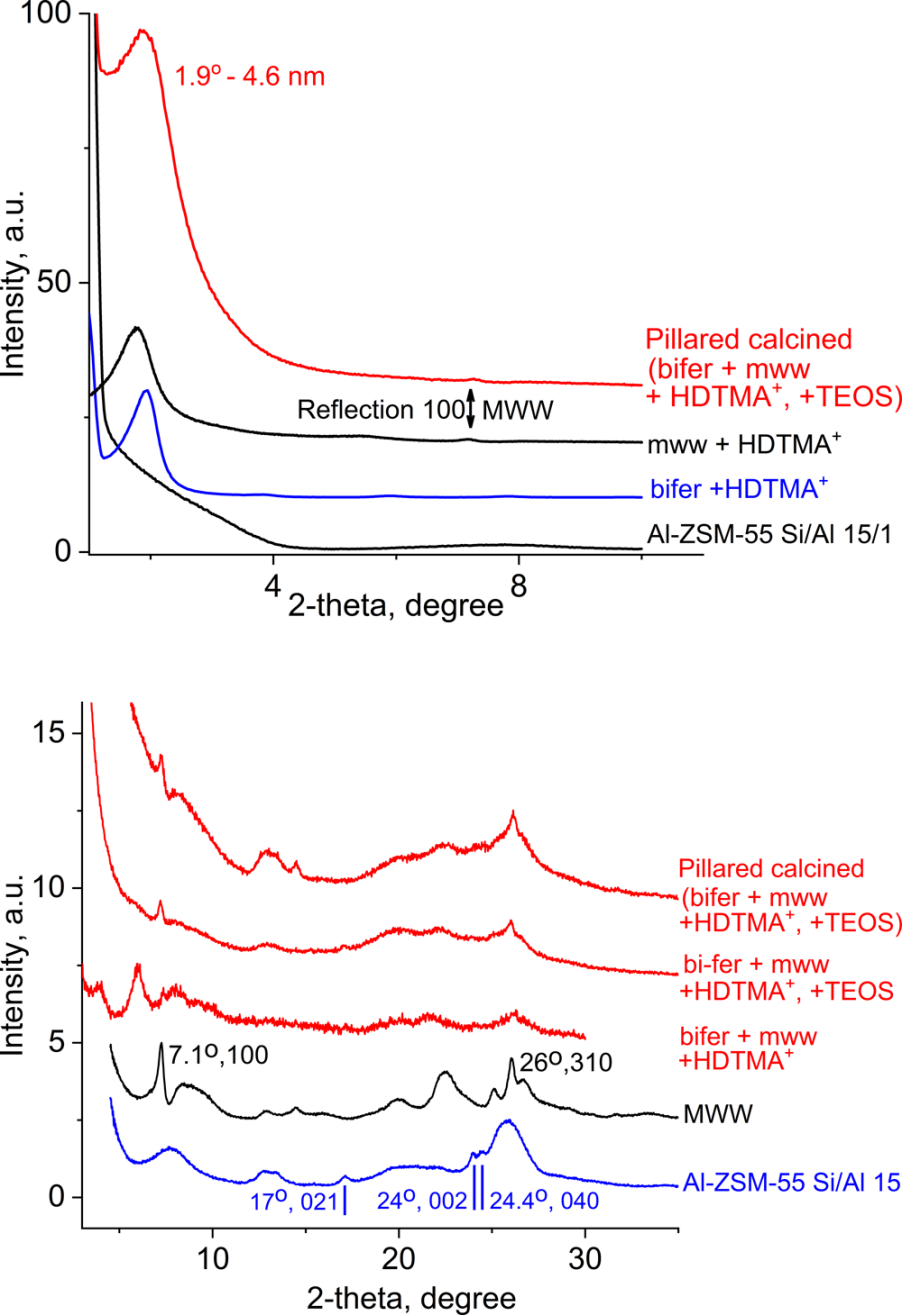
XRD patterns for pillared mixed zeolite material composed of mww and bifer layers. Top: low-angle region including mww and bifer materials separately flocculated with HDTMA^+^. Bottom: higher-angle region with marked intralayer reflections identifying mww and bifer layers.

The mixing of solution of mww and bifer layers produced homogeneous liquids without visible precipitation/flocculation. Solid products have been isolated by addition of flocculants like alcohol or the cationic surfactant HDTMA-Cl. The latter afforded surfactant swollen multilayered materials with expanded *d*-spacing above 4.5 nm (as-synthesized, not shown) and at 4.6 nm after pillaring with TEOS and calcination (Figure 7).

Both mww and bifer exhibit similar *d*-spacing values individually with HDTMA, also shown in Figure 7. This raises the question whether the mww and bifer sheets are randomly interstratified (mixed at the unit cell level) or segregated (restacked individually). The question was difficult to answer even with TEM because the layers could not be distinguished unambiguously based on both thickness and the presence of pores, seen as white lines in the center. This subject requires more elaborate studies of both the synthesis, including different zeolite ratios, and characterization. In the present case, both the mww and bifer layers could be observed in the powder XRD but the scattering from the former was much stronger. Thus, in order to make the bifer layers better visible for the proof-of-principle demonstration, the relative amounts of both zeolites were chosen as ca. 1:3–4 (mww to bifer).

Despite large excess of the bifer, the reflections of the MWW structure were still more pronounced in the XRD (Figure 7 bottom). The pattern contained only a few distinct features, mainly broad, but included the intralayer MWW reflections 100 and 310 at ca. 7 and 26° 2θ. The presence of both layers can be detected in the XRD pattern of the silica pillared product obtained by treatment with TEOS and calcination. The mww layers are identified based on relatively sharp and distinct intralayer 100, 200, and 310 reflections, and the broad band starting at 8° 2θ. The bifer features are seen at ca. 13° 2θ (broad), 17° 2θ (before calcination), and the doublet at 24° 2θ (merged, broad). The bifer features appear easier to observe before calcination, which seems to cause deformation and result in weaker XRD scattering.

**Figure 8:**
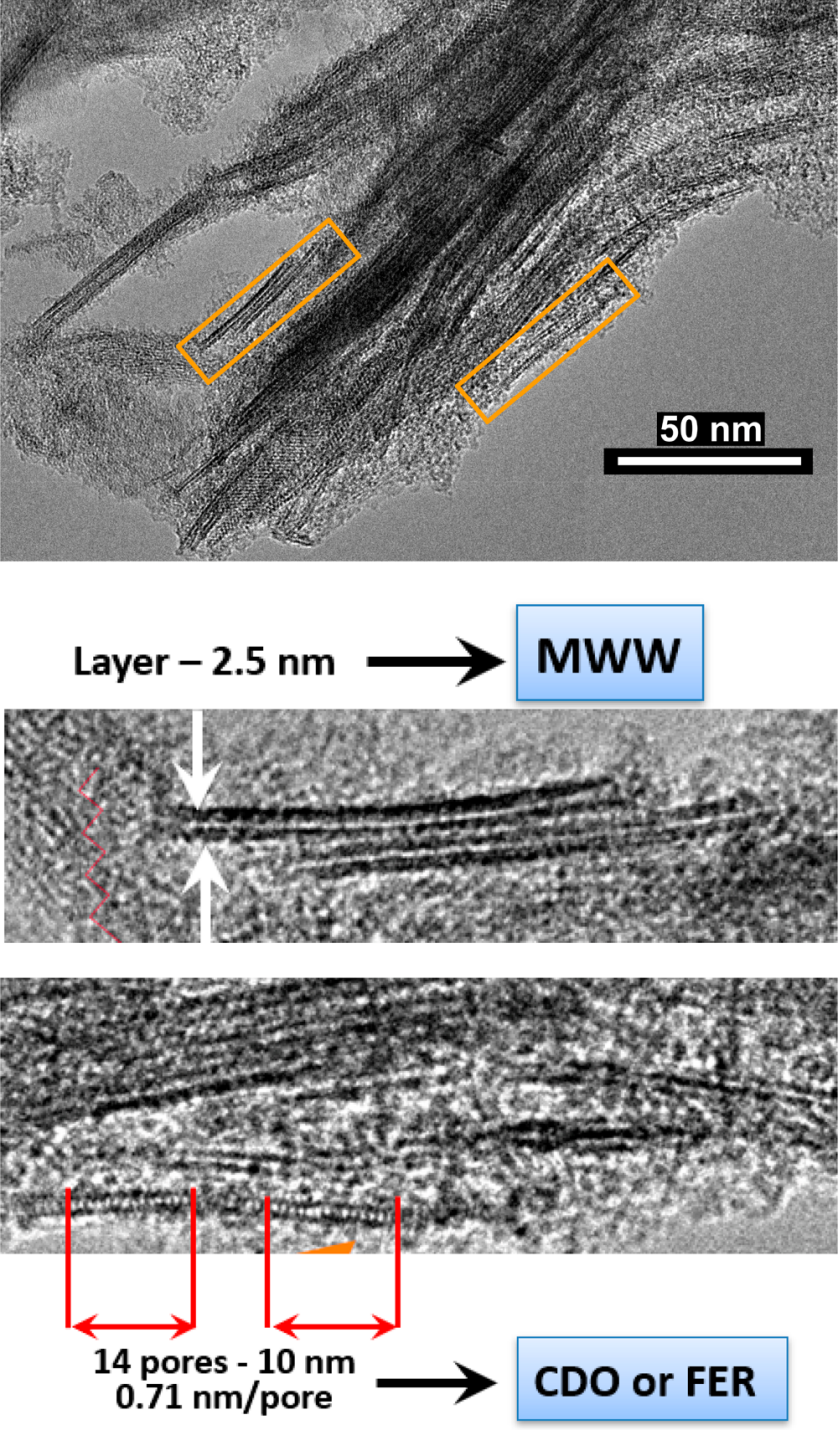
TEM image of the silica pillared mixed zeolite material, mww with bifer. Top: overall. Middle and bottom: selected fragments to highlight individual layer structures.

The mixed zeolite nature and pillared multilayer structure were confirmed by TEM of the calcined TEOS pillared product. The TEM image in Figure 8 shows layers with 2–2.5 nm thickness, mostly separated by comparable 2–3 nm distances, somewhat variable. Despite uncertain differentiation between mww and bifer layers based on thickness and pores in the middle, in many instances, the assignment is possible with high level of confidence, as illustrated by selected magnified fragments. Remarkably, one of the views allows observation of 8-MR pores, recognized by their density/spacing (Figure 8, bottom). Ironically, both FER and CDO structures possess such pore arrangements, so even this image does not allow definite identification.

**Figure 9:**
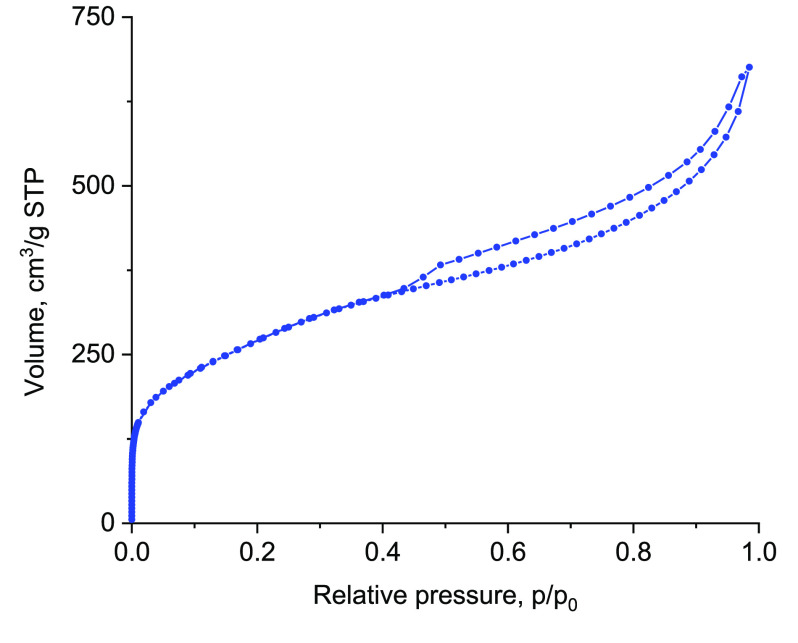
Nitrogen adsorption/desorption isotherm for the pillared mww–bifer hybrid material.

The enhanced pore structure and textural properties of the pillared materials are confirmed by its nitrogen adsorption. The isotherm shown in Figure 9 is typical for pillared layered materials. The product had BET area equal to 998 m^2^/g and total pore volume of 1.05 cm^3^/g. The acid site concentrations, Brønsted −200 μmol/g and Lewis −100 μmol/g, are lower than in pure zeolites due to addition of silica as pillars. Remarkably, the catalytic activity in mesitylene benzylation (Figure S10) is the same as that of the active component mww alone despite its content being <30% (estimated upper limit, e.g., based on acid site concentration). Clearly, the composite is better than the sum of its components, showing benefits from such materials. Full exploitation and explanation will require more elaborate studies, but the prospects are promising in practice and interesting fundamentally.

## Summary and Conclusions

This study extends liquid exfoliation to the second layered zeolite material, designated Al-ZSM-55, with ferrierite-related structure, proving formation of unilamellar nanosheets by treatment with TBAOH. The layers have rectangular unit-cell dimensions, similar to the fer layers but twice the approximate layer thickness, ca. 2 nm. They were designated bifer and were synthesized by substituting boron with aluminum in the preparation of the layered zeolite precursor ZSM-55 containing originally 0.9 nm thick fer layers and choline template. The evidence of monodispersity of the layer thickness and the internal structure was confirmed by five characterization techniques. The layers show strong acid sites typical for zeolites and 8-MR pores in the layers and are active in the benzylation of mesitylene as a catalytic test reaction for accessibility to bulky molecules. The detailed data related to the layered structures could not allow definite distinction between two possible topologies CDO and FER that can be made from fer layers. The chemical rationale based on the presence of Al in the synthesis points to the latter as more probable.

The suspensions of bifer layers were used to prepare surfactant intercalated composites and unprecedented mixed zeolite hierarchical structures comprising mww and bifer layers. This appears to be the first example of zeolitic layered materials composed of two different topologies obtained by mixing postsynthetically the suspensions, with preservation of intrinsic features of both layer types. This approach may allow preparation of materials with activity and functions tailored by combining selected frameworks.

So far, no 3D framework based on the bifer layers has been obtained: neither through condensation of a multilayered precursor, which is unavailable/unknown, nor by reassembly of the exfoliated layers from solutions. This lack of a 3D reference seriously hinders more accurate structural characterization of the bifer layers, temporarily or in general, but does not hinder seriously the development of the exfoliation side. Layer reassembly from solution into an ordered structure will be difficult because of the disparate sizes and shapes of the exfoliated layers and together with synthesis of an ordered precursor remain as challenges for the future.

## Experimental Section

### Syntheses of Al-ZSM-55 and Derivative Compounds

#### A Al-ZSM-55 with Si/Al Equal to 60/1

The synthesis mixture contained 9.3 g of Aerosil, 0.96 g of aluminum nitrate nonahydrate, 3.7 g of 50% NaOH, 9.42 g of choline chloride, and 36.7 g of water. All reagents were from Sigma-Aldrich. The corresponding molar ratios Si:Al:Na_2_O:choline:water were 1:0.016:0.15:0.44:14.1. The mixture was homogenized by rotation overnight in a Teflon lined 100 mL autoclave and heated with rotation for 192 h at 143 °C. The solid product was isolated by filtration, washed with water, and air-dried. The yield was ca. 11 g.

#### B Al-ZSM-55 with Si/Al 15.7/1

The mixture was prepared using 16.2 Ludox LS30, 0.5 g of sodium aluminate (40–46% Na_2_O, 50–56% Al_2_O_3_, Riedel-de Haën), 2.5 g of 50% NaOH, 10 g choline chloride, and 30 g of water. The Si:Al:Na_2_O:choline:water molar ratio was equal to 1:0.064:0.23:0.88:30. After overnight rotation at room temperature, the synthesis was carried out at 150 °C for 120 h. The product was isolated as above.

Powder XRDs of A and B differed significantly because A was dominated by the pattern of ZSM-55, whereas B was pure bifer. The isolated exfoliated layers were identical based on XRD.

### Template Extraction (Partial)

Illustrative conditions: 108 g of methanol (>99.8%, Riedel-de Haën), 11 g of concentrated HCl (36–39%, Avantor, Poland), and 3.15 g of Al-ZSM-55 were stirred at 50 °C for 24 h. The solid, designated H-Al-ZSM-55, was filtered, washed with methanol, and dried in air.

### Exfoliation in TBAOH Solutions

The general procedure involved stirring 0.5 g of Al-ZSM-55 or H-Al-ZSM-55 with 5 or 10% TBAOH solution (13–30 g) for 1–2 h, centrifugation at 10 000 rpm for 20 or more minutes (depending on the settling of solids), decantation of clear liquid, and addition of water to obtain additional solutions of nanosheets by stirring (including overnight) and centrifugation. The solutions were denoted as supernatants 1, 2, etc.

### Preparation of Organic Intercalated Composites

The solutions of TEAOH (20%) and HDTMA-Cl (25%), both from Sigma-Aldrich, were added to bifer solutions after centrifugation in ratios 1:1–4 producing abundant white solid instantly. The isolation involved centrifugation, washing with water (1–2 times), drying at 50–80 °C.

### Preparation of the Mixed Mww/Bifer Zeolite Material

0.26 g of MCM-56^1^ was stirred with 13 g of 5% TBAOH and 0.485 g of Al-ZSM-55 (preparation B) was stirred with 27 g of 5% TBAOH, for 1.5 h. Following centrifugation for 40 min at 10 000 rpm, clear liquids were decanted and that from Al-ZSM-55 poured into the MCM-56 solution. After brief stirring of the mixture, the obtained clear yellowish liquid with no visible precipitate, was allowed to stand for overnight. 25 g of the liquid was pipetted out and combined with 48.5 g of 25% HDTMA-Cl solution. The solid was isolated by centrifugation at 10 000 rpm for 10 min, washed with water, centrifuged out again, and dried in air. It was mixed with 20 g of TEOS, stirred overnight at room temperature and again isolated by centrifugation at 10 000 rpm for 10 min and drying in air for overnight and then at 60 °C. Subsequent calcination was carried out at 540 °C, ramping up at 2 °C/min.

### Freeze-Drying (Lyophilization)

Lyophilization (freeze-drying) was carried out using Labconco FreeZone and Shell Freezer. Samples were frozen for 20 min at −42 °C with rotation. The frozen samples were maintained under vacuum (<0.1 mbar) at −49 °C until complete removal of H_2_O and volatile components.

### Characterization

The detailed procedures were also reported elsewhere.^1^

#### Atomic Force Microscopy (AFM)

The reagents used for sample preparation were obtained from the following sources: branched polyethylenimine (PEI, molecular weight ∼600 g/mol) was purchased from Sigma-Aldrich; toluene (p.a.), THF (p.a.), ethanol (p.a.), NaCl (p.a.), H_2_SO_4_ (96%), and H_2_O_2_ (30%) were purchased from Chempur. All regents were used as received.

Silicon wafers were sonicated in ethanol for 10 min, dried, and placed in piranha solution (H_2_SO_4_:H_2_O_2_, 3:1) for 15 min at room temperature. Afterward, the plates were rinsed with copious amounts of deionized water, THF, and toluene, and dried in the stream of argon. Such prepared substrates were then treated with hand-held atmospheric plasma cleaner (Plasma Wand, Plasma Etch Carson City, Nevada, USA) for 60 s and subsequently placed in the glass vials with polyethylenimine solution (PEI, 600 g/mol, 1 g/L) in 0.01 M NaCl. PEI deposition was supported by pulse sonication (15 min). After completion, the samples were rinsed with copious amount of deionized water and dried in the stream of argon. A solution obtained from the Al-ZSM-55 preparation A diluted 100 times was spin-casted (2 000 rpm, 120 s) on the PEI-modified support.

Atomic force microscopy (AFM) images were obtained with a Dimension Icon AFM (Bruker, Santa Barbara, CA) working in the PeakForce Tapping (PFT) and QNM modes. TESPA (Bruker) probes with a nominal spring constant of 42 N/m were used for all measurements. Total surface coverage and percentage of single layer structure (image area occupied by single layer structure/total surface area covered with sample) was calculated using bearing analysis. The images were captured in 8 different places on the sample (resolution 384 × 384, size 5 × 5 μm^2^ for bearing analysis and 2 × 2 μm^2^ for analysis of topography, see Figure 1).

#### In-Plane XRD

Solutions of bifer layers were obtained from the Al-ZSM-55 preparation A treated with 10% TBAOH and centrifugation at 10 000 rpm for 10 min. The nanosheets were deposited on a Si substrate via the Langmuir–Blodgett process. The substrate surface was substantially covered by a monolayer film of 2D nanosheets. XRD data were recorded in the in-plane mode using synchrotron radiation X-rays (λ = 0.11988(2) nm) at Photon Factory, BL-6C, KEK. The raw data showing a strong background at a low angular range were processed by baseline removal and peaks having fwhm ∼0.2 degree were selected and all peaks were indexed based on a 2D rectangular unit cell (1.463 × 0.746 nm^2^) by applying *Appleman* software.^35^

#### In Situ XRD Measurements

Solutions were obtained from the preparation A and 10% TBAOH. The sample preparation involved centrifugation of the colloidal suspension at 20 000 rpm for 60 min, which separated the dispersed materials from majority of the liquid. It afforded a glue-like sediment at the bottom and a clear solution at the top. The former was loaded onto a XRD sample holder and the XRD data were measured at a relative humidity (RH) of 95% using a specially designed diffractometer (RINT-Ultima) to avoid sample drying (see Figure S2 in the Supporting Information in ref (1)). The RH in the sample chamber of the diffractometer was regulated by circulating dry and moisture-saturated N_2_ gas at a specified ratio. XRD measurement was conducted using graphite monochromatized Cu Kα radiation (λ = 0.15405 nm) with steps of 0.020 and scan speed of 10 scans/min.

As required by the procedure, the layer structure factors were calculated using the equation, given below, based on the doubled fer layer with both CDO and FER topologies.
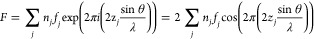
where*f*_*j*,_ θ, and λ are atomic scattering factors, diffraction angles, and X-ray wavelength, respectively. The atomic positions along the layer normal are *z*_*j*_.

The square of the layer structure factor should correspond to the scattering from the aggregate of 2D crystallites that lie parallel to the sample holder.

#### TEM of bifer Layers

A TBAOH solution of the bifer nanosheets was dropped onto a carbon film coated grid and then baked at 150 °C in air for half an hour before TEM observations. The sample was extremely sensitive to electron irradiation. To reduce irradiated induced damage, an aberration-corrected JEM ARM200F microscope operated at 80 kV was used to examine the morphology and cross-sectional structure. An FEI Titan Themis Z microscope with a fast Gatan K2 direct detection camera was operated at 300 kV in low-dose mode to characterize the in-plane structure. A high-resolution TEM image of the in-plane structure was obtained using an average background subtraction filter (ABSF) to enhance the contrast of the crystalline structure.^46^ The fast Fourier transform (FFT) pattern showed perpendicular reflections, which are close to (002) and (040), respectively.

#### TEM of the Pillared mww/bifer Composite

TEM imaging was performed using JEOL NEOARM200F at accelerating voltage of 200 kV. The microscope was equipped with a Schottky-type FEG and TVIPS XF416 CMOS camera. The alignment was performed by a standard method using a carbon film covered with gold nanoparticles. The electron dose was kept below a current density of 3 pA/cm^2^ because of the low beam stability of the samples.

#### Basic Characterization by X-ray Powder Diffraction, Nitrogen Adsorption, XRF, and FTIR

Powder XRD patterns were collected using a Bruker AXS D8 Advance diffractometer equipped with a graphite monochromator and a position sensitive detector (Våntec-1) in Bragg–Brentano geometry and a Rigaku MiniFlex diffractometer in reflection mode. The radiation used was CuKα with λ = 0.154 nm with typical XRD step size equal to 0.02°.

Nitrogen adsorptions were carried out by the standard method at −196 °C (liquid nitrogen temperature) in an ASAP 2025 (Micromeritics) static volumetric apparatus. The samples were outgassed at 350 °C using a turbomolecular pump to remove adsorbed water before exposure to the sorbent gas.

The content of Al and Si was evaluated by XRF with samples formulated into pellets, 20 mm in diameter, with the use of energy-dispersive XRF spectrometer (Thermo Scientific, ARL QUANT’X). The X-rays of 4–50 kV (1 kV step) with a beam size of 1 mm were generated with the Rh anode. The detector used was a 3.5 mm Si(Li) drifted crystal with a Peltier cooling (ca. −88 °C). *UniQuant* software was used for quantitative analysis based on calibration with a series of metallic standards.

The concentration of Lewis (LAS) and Brønsted (BAS) acid sites was determined by adsorption of probe molecules: ammonia, pyridine (Py) and pivalonitrile (PN) followed by IR spectroscopy using Tensor 27 from Bruker, MTC detector, spectral resolution 2 cm^–1^. Zeolites were pressed into self-supporting wafers with a density of ca. 8 mg/cm^2^ and activated in situ at 450 °C for 1 h at high vacuum (1 × 10^–5^ mBar). Excess of pyridine and pivalonitrile vapors or ammonia (ca. 25 mbar equilibrium pressure) were adsorbed at 170 °C (pyridine) 100 °C (ammonia) or 25 °C (pivalonitrile) followed by desorption for 20 min at the adsorption temperature. Spectra were recalculated to a wafer mass equal 10 mg. Concentration of Lewis (LAS) and Brønsted (BAS) acid sites were evaluated from the intensities of bands at 1454 cm^–1^ (LAS) and at 1545 cm^–1^ (BAS) using absorption coefficients determined earlier in our laboratory using external standards,^48^ ε(LAS) = 0.165 cm^2^/μmol, and ε(BAS) = 0.044 cm^2^/μmol, and the intensities of corresponding pyridine maxima after pyridine desorption at 170 °C to ensure complete removal of weakly adsorbed species.

### Catalytic Testing

#### Catalyst Preparation

Samples calcined at 540 °C for 3–6 h were converted into NH_4_^+^-form by contacting with 1 M solution of NH_4_NO_3_ (Avantor Poland, p.a.) for 1 h at room temperature (>20:1 v/w, e.g., 20 mL of solution per 0.5 g of zeolite), repeated two to three times, filtered, washed with deionized water, dried, and activated at 450 °C for 5 h.

#### Catalytic Tests

**Figure 10:**
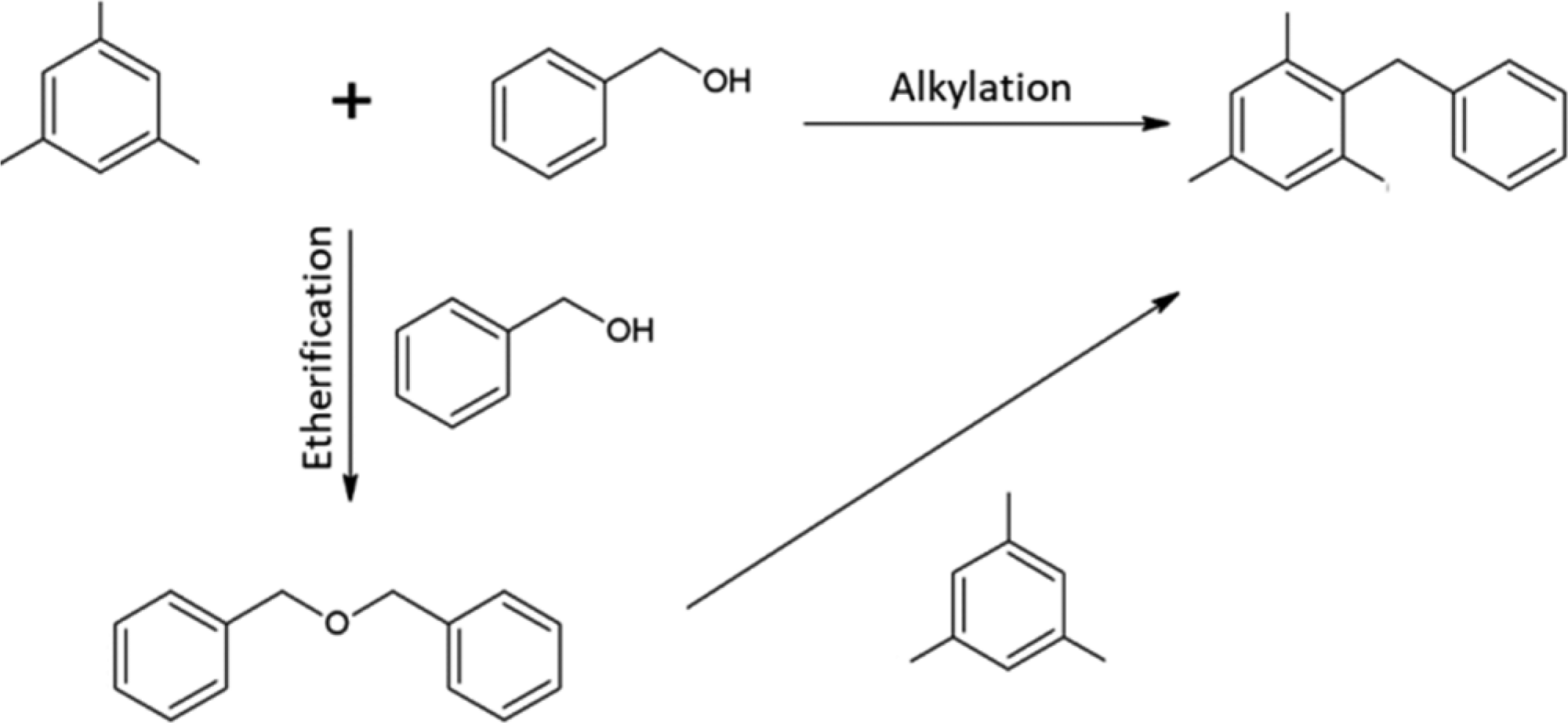
Alkylation of mesitylene with benzyl alcohol.

The test reaction–liquid phase benzylation of mesitylene with benzyl alcohol (Figure 10) was carried out in three-neck round-bottom flasks attached to a reflux condenser with heating in a multiexperiment workstation StarFish (Radleys Discovery Technologies) under atmospheric pressure at 80 °C. The reaction was started (0.0 time) by addition of 0.2 g of benzyl alcohol to the mixture of mesitylene (19.0 g), 50 mg of a catalyst, and dodecane (0.1 g) as an internal standard that was preheated for 30 min at the reaction temperature. Liquid samples were withdrawn at regular intervals and analyzed in the gas chromatograph Agilent 7820A GC with an FID detector using a 30 m packed DB-5 column. The conversion of alcohol was calculated according to the formula:
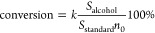
where *S* is the area of respective peak in the chromatogram, *k* is the calibration coefficient (mol), and *n*_0_ is the starting amount of alcohol (mol). The reaction can proceed by the routes shown in Figure 10.

## Supporting Information Available

AFM of thicker particles, powder XRDs of bifer materials recovered by lyophilization and at various temperature to 540 °C with listing of peak positions, simulated XRDs for FER and CDO layers, organic analysis of selected samples, square of structure factors calculated based on fer layer and FER structures, detailed SEAD analysis, edge-on and additional TEM of HDTMA-bifer and pillared mww+bifer images, catalytic testing–benzylation of mesitylene (PDF)
