# Supplementary material for: Exfoliated Ferrierite-Related Unilamellar Nanosheets in Solution and Their Use for Preparation of Mixed Zeolite Hierarchical Structures
Source: J Am Chem Soc. 2021 Jul 15;143(29):11052–62. doi: 10.1021/jacs.1c04081 (PMC8397323; doi:10.1021/jacs.1c04081)
Supplement: Supplementary file 1 — ja1c04081_si_001.pdf [file ja1c04081_si_001.pdf]

# Supporting Information

# Exfoliated ferrierite-related unilamellar nanosheets in solution and their use for preparation of mixed zeolite hierarchical structures

Wieslaw J. Roth*,[1] Takayoshi Sasaki*,[2] Karol Wolski,[1] Yasuo Ebina,[2] Dai-Ming Tang,[2] Yuichi Michiue,[2] Nobuyuki Sakai,[2] Renzhi Ma,[2] Ovidiu Cretu,[4] Jun Kikkawa,[4] Koji Kimoto,[4] Katarzyna Kalahurska,[1] Barbara Gil,[1] Michal Mazur,[3] Szczepan Zapotoczny,[1] Jiri Čejka,[3] Justyna Grzybek,[1] Andrzej Kowalczyk[1]

[1]Jagiellonian University, Faculty of Chemistry, Gronostajowa 2, 30-387 Kraków, Poland

[2]International Centre for Materials Nanoarchitectonics (WPI-MANA), National Institute for Materials Science (NIMS), 1-1 Namiki, Tsukuba, Ibaraki 305-0044, Japan.

[3]Department of Physical and Macromolecular Chemistry, Faculty of Science, Charles University, Hlavova 8, 12840 Prague 2, Czech Republic

[4]Research Center for Advanced Measurement and Characterization, National Institute for Materials Science, 1-1 Namiki, Tsukuba, 305-0044, Japan

Contents


| | |
|---|---|
| **Figure S1** | AFM results showing thicker particles from the bifer solutions (estimated content <6%). |
| **Figure S2** | XRD patterns of solids isolated from 3 solutions of exfoliated bifer layers by lyophilization. |
| **Figure S3** | Variable temperature powder XRD patterns for bifer layers isolated by lyophilization heated from room temperature to 540°C. |
| **Table S1** | Analysis of the variable temperature powder XRD patterns. |
| **Table S2** | Elemental analyses for C, H and N in selected samples. |
| **Figure S4** | Simulated powder XRD patterns for single bifer layers assuming FER and CDO structure. |
| **Figure S5** | Combined in-plane and powder XRD patterns. |
| **Figure S6** | Square of structure factors calculated based on single fer layer and double-layer FER structure. |
| **Figure S7** | Detailed Electron Diffraction analysis including weak data. |
| **Table S3** | Comparison of cell dimensions – in-plane and ED. Values similar within accuracy of ED. |
| **Figure S8** | Edge-on TEM views with pores compared with the atomic model and simulated TEM images. |

**Figure S9** Additional TEM images – flocculated HDTMA surfactant – bifer composite; another view of the mixed mww-bifer pillared material.

**Figure S10** Catalytic testing – alkylation of mesitylene with benzyl alcohol catalyzed by different materials obtained from monolayer zeolite solutions.

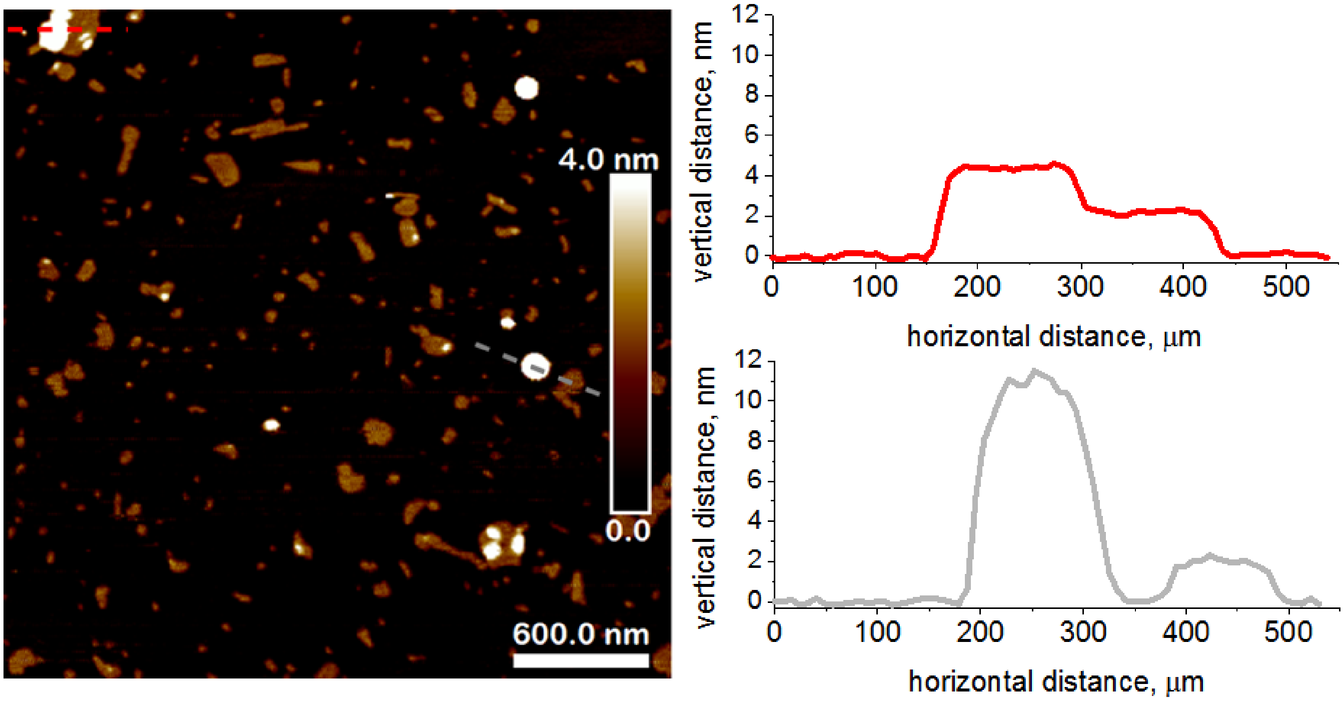


**Figure S1.** AFM results showing thicker particles from the bifer solutions (estimated content <6%).

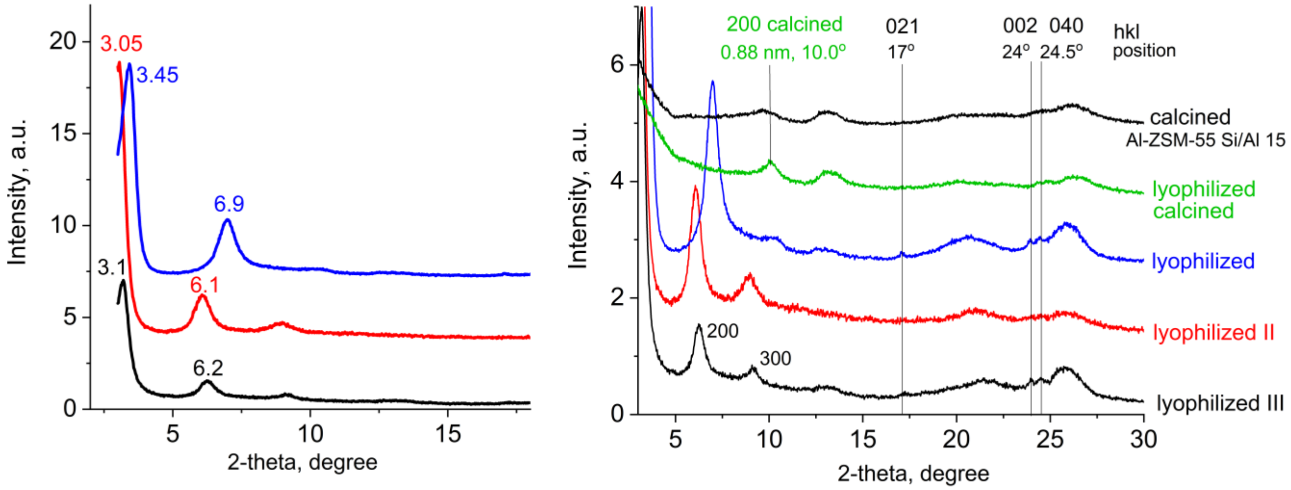


**Figure S2**. XRD patterns of powdered solids isolated from 3 solutions of exfoliated bifer layers by lyophilization. Left – lower angle highlighting interlayer reflections and their slightly variable positions. Right – positions of identified invariant interlayer reflections with indices. The calcined samples show contracted d-spacing and layer thickness around 1.76 nm and deterioration of quality (diminished scattering intensities and peak sharpness).

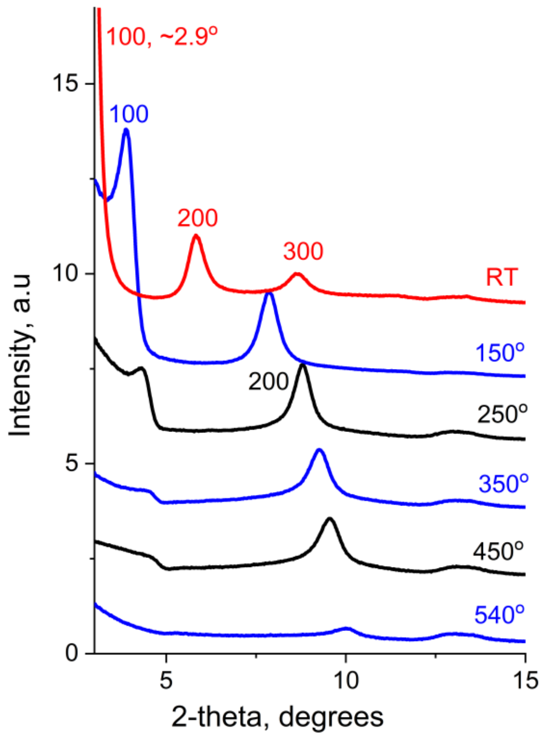


**Figure S3.** Variable temperature powder XRD patterns for bifer layers isolated by lyophilization heated from room temperature to 540 °C showing contraction of the structure due to removal of intercalated compounds and shifting of interlayer reflections to higher $2\theta$ angles (marked selectively, highlighted on the left) with preservation of intralayer reflections, highlighted on the right.

**Table S1**. Analysis of the variable temperature powder XRD patterns. The listed positions are nominal, i.e. maxima from the intensity vs. 2-theta lists. The most intense peaks labeled in red.

| Heat treatment, °C | Peak maxima, nominal, 2-theta degrees | | | | | | | | weight left, % |
|---|---|---|---|---|---|---|---|---|---|
| | Miller indices | | | | | | | | |
| | 100 or unknown | 200 | 300 | 400 | 021 | 031 | 002 | 040 | |
| 25 | **2.8-2.9*** | 5.82 | 8.60 | 11.46 | 17.02 | 21.82 | 23.90 | 24.36 | |
| 150 | **3.88** | 7.86 | | | 17.12 | 22.08 | 24.10 | 24.58 | 65.1 |
| 250 | 4.34 | **8.80** | | | 17.16 | 21.92 | 24.04 | 24.46 | 56.1 |
| 350 | 4.3 sh** | **9.26** | | | 17.22 | 22.02 | 24.12 | 24.64 | 52.6 |
| 450 | 5.5 sh** | **9.54** | | | 17.26 | 21.98 | 24.20 | 24.68 | 50.0 |
| 540 | 5.3 asym*** | **10.00** | | | 17.26 | 22.04 | 24.32 | 24.74 | |

*extrapolated;**shoulder;***asymmetric

**Table S2.** Elemental analyses for C, H and N in selected samples.

| Material | Weight % | | | Molar ratio | | Estimated % organic |
|---|---|---|---|---|---|---|
| | N | C | H | C/N | H/N | |
| As-synthesized Al-ZSM-55 | 2.41 | 10.18 | 3.334 | 4.93 | 19.4 | 16% choline (C/N=5) |
| Exchanged 1 M $NH_4NO_3$, 2x, RT | 1.56 | 5.56 | 2.126 | 4.16 | 19.1 | 9% choline + $NH_4^+$ |
| Solid from lyophilization | 2.36 | 27.23 | 5.609 | 13.46 | 33.3 | 35% $N(C_4H_9)_4^+$ |

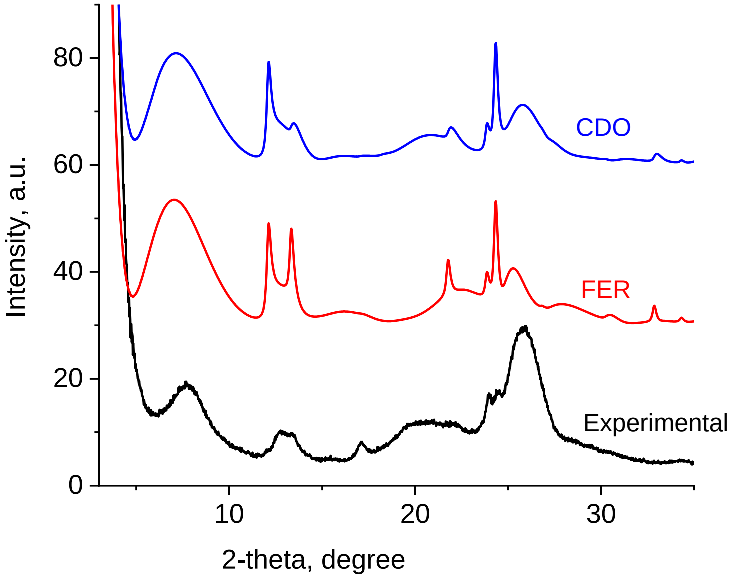


**Figure S4.** Simulated powder XRD patterns for single bifer layers assuming FER and CDO structure without optimization of atomic positions, obtained using program *Mercury* 2020.1.

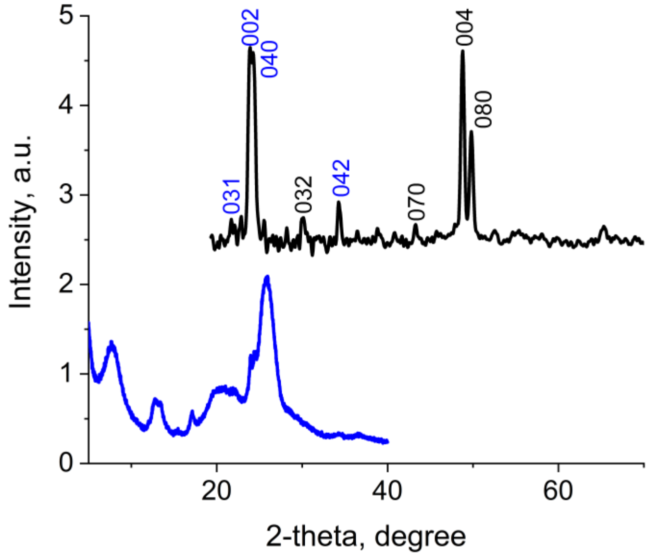


**Figure S5.** Combined in-plane and powder XRD patterns. Matching 0kl reflections marked in blue.

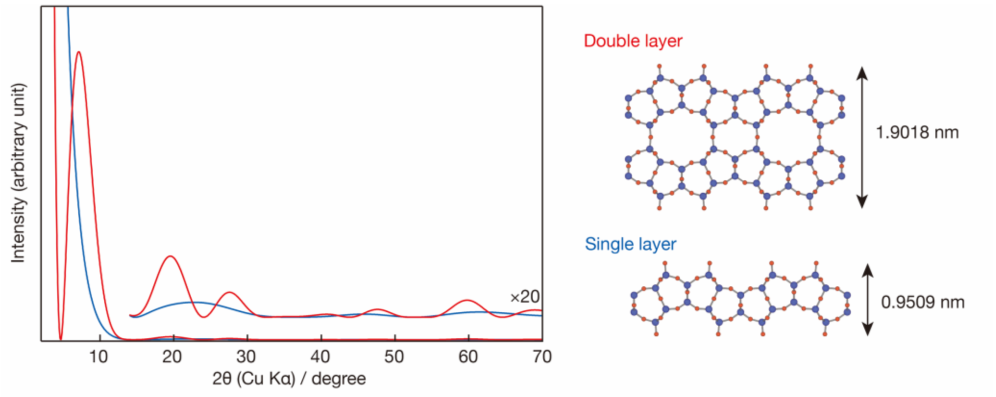


**Figure S6.** Square of structure factors calculated based on single-layer (blue) and double-layer (red) FER structure. The square of structure factor for the single-layer FER shows rather broad and featureless profile when compared with that for the double-layer FER, which is obviously more similar to the observed pattern (Figure 4 in the manuscript).

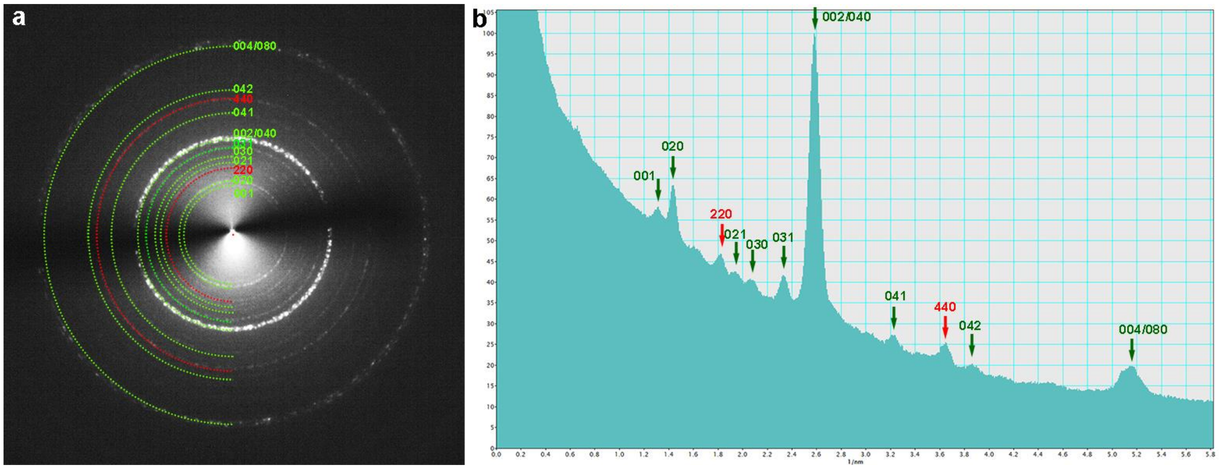


Ring indexing order (right) and *d*-spacing including interlayer reflections marked in red.

| Number | a | b | c | ***d*-spacing, nm** exp. | calc. | error, % |
|---|---|---|---|---|---|---|
| 1 | 0 | 0 | 1 | 0.764 | 0.756 | -1.08 |
| 2 | 0 | 2 | 0 | 0.695 | 0.705 | 1.44 |
| 3 | 2 | 2 | 0 | 0.549 | 0.563 | 2.48 |
| 4 | 0 | 2 | 1 | 0.517 | 0.515 | -0.36 |
| 5 | 0 | 3 | 0 | 0.483 | 0.470 | -2.66 |
| 6 | 0 | 3 | 1 | 0.429 | 0.403 | -6.06 |
| 7 | 0 | 0 | 2 | 0.387 | 0.378 | -2.40 |
| 8 | 0 | 4 | 1 | 0.311 | 0.319 | 2.70 |
| 9 | 4 | 4 | 0 | 0.275 | 0.282 | 2.48 |
| 10 | 0 | 4 | 2 | 0.259 | 0.258 | -0.61 |
| 11 | 0 | 0 | 4 | 0.194 | 0.189 | -2.74 |

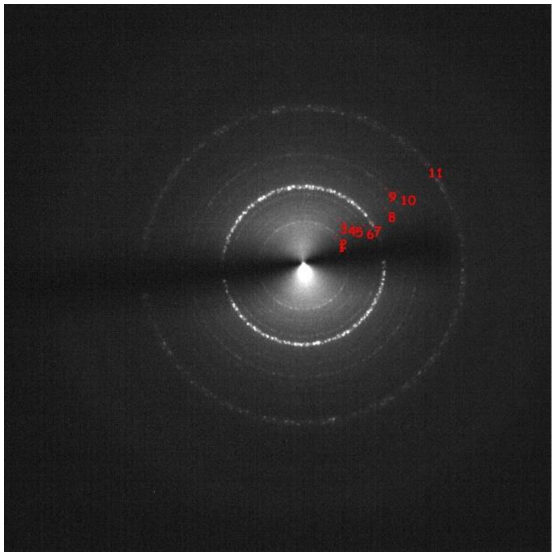


**Figure S7.** SAED with detailed indexing (top) and listing of the rings (bottom). Most of the rings are indexable as in-plane reflections. There are 2 rings (indicated in red in the table, bottom left) that could not be indexed as in-plane peaks. This may suggest that the sample contained unexfoliated phase contaminants. The amounts are small or trace level, considering their weak intensity.

**Table S3.** Comparison of cell dimensions – in-plane and ED. Values similar within accuracy of ED.

| | ***d*-spacing, nm** | |
|---|---|---|
| axis | ED | in-plane |
| a | 1.8744 | 1.76* |
| b | 1.4099 | 1.4638 |
| c | 0.7557 | 0.7461 |

* calcined

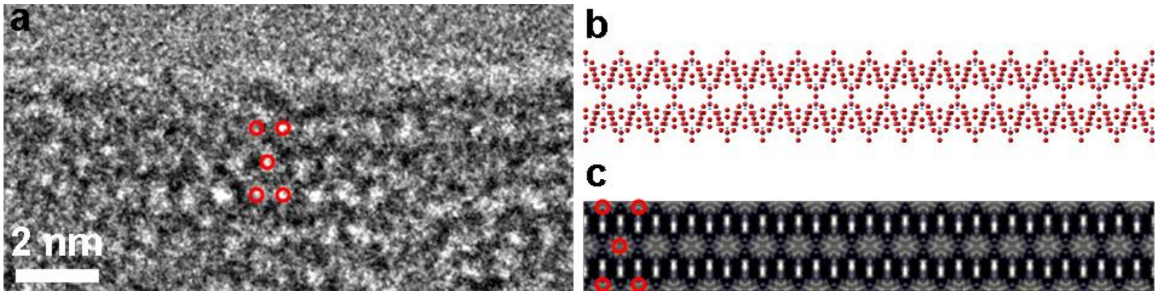


**Figure S8a**. Side-view TEM image (a), atomic model (b), and simulated TEM image (c) with features consistent with CDO along [001] zone axis or FER along [010] zone axis.

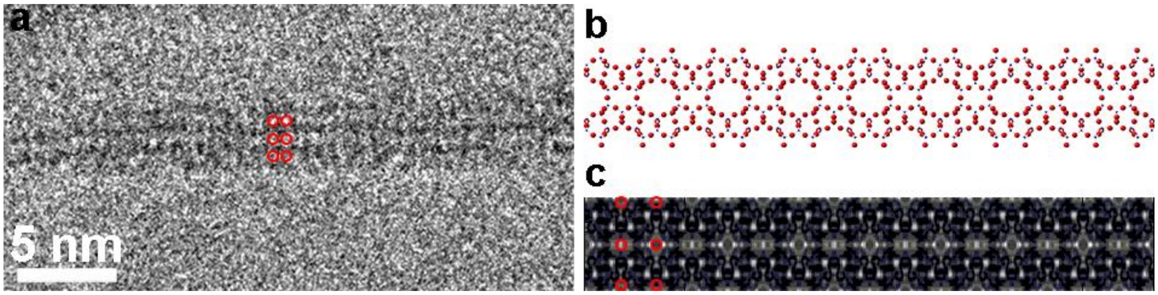


**Figure S8b**. Side-view TEM image (a), atomic model (b), and simulated TEM image (c) with features consistent with FER along [001] zone axis.

**Figure S8.** TEM views in the edge-on positions with the pores (atoms) aligned along with the crystalline axis and also parallel to the electron beam that we were able to acquire with enough high quality to enable comparison with the atomic model and simulated TEM images. The top image shows that the pore positions are arranged in a face-centered pattern, consistent with CDO along [001] zone axis or FER along [010] zone axis. The lower figure shows a side-view TEM image where the pores are arranged into a rectangle pattern, that is consistent with FER [001] along zone axis.

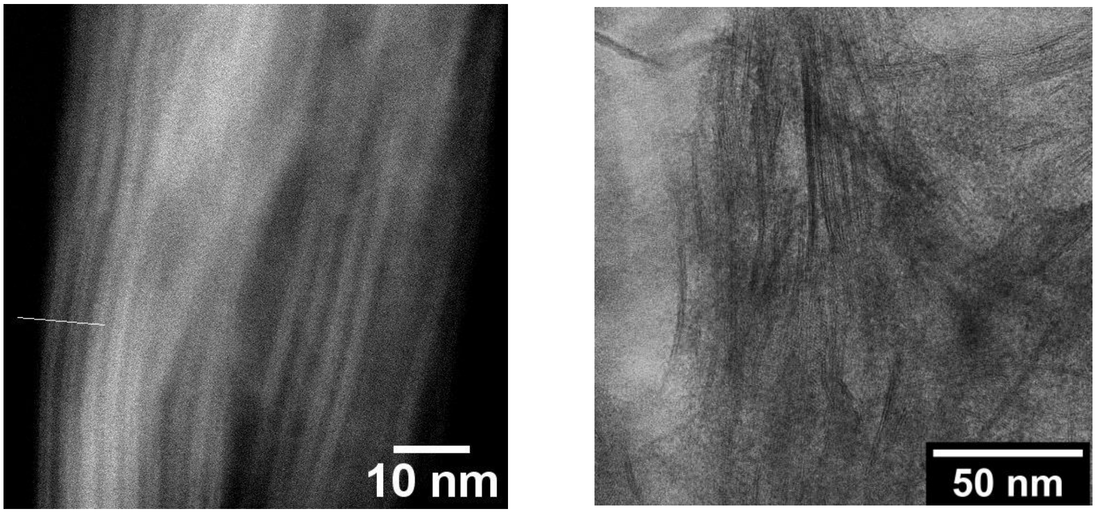


**Figure S9**. Additional TEM images – left, flocculated HDTMA surfactant bifer composite; right – another view of the mixed mww-bifer pillared material.

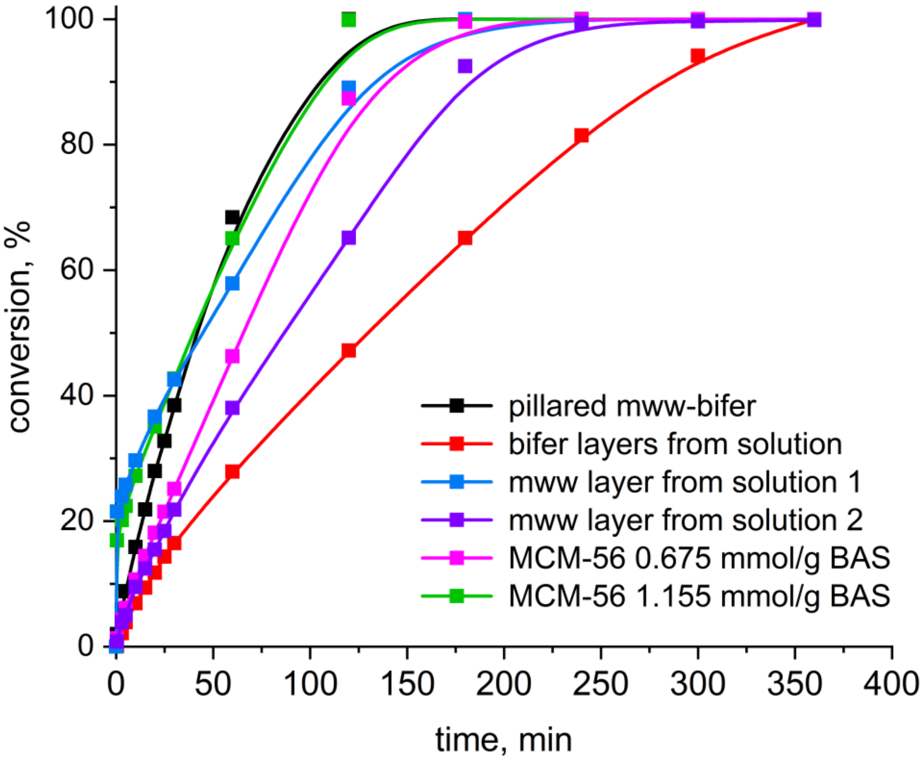


**Figure S10**. Catalytic testing – alkylation of mesitylene with benzyl alcohol catalyzed by different materials obtained from monolayer zeolite solutions; includes zeolites MWW isolated from solutions of mww layers and the starting MCM-56 with different BAS concentrations.
